# Symptomatic Remission and Counterfactual Reasoning in Schizophrenia

**DOI:** 10.3389/fpsyg.2016.02048

**Published:** 2017-01-06

**Authors:** Auria Albacete, Fernando Contreras, Clara Bosque, Ester Gilabert, Ángela Albiach, José M. Menchón

**Affiliations:** ^1^Department of Psychiatry, Bellvitge University Hospital-IDIBELLBarcelona, Spain; ^2^Department of Clinical Sciences, School of Medicine, University of BarcelonaBarcelona, Spain; ^3^Carlos III Health Institute, Centro de Investigación Biomédica en Red de Salud Mental (CIBERSAM)Barcelona, Spain; ^4^Fundació per a la Investigació i Docència María Angustias Giménez, Germanes HospitalàriesBarcelona, Spain; ^5^Mental Health Unit L'Hospitalet, SAP Delta Llobregat, AP Costa de Ponent, Catalan Institute of HealthBarcelona, Spain

**Keywords:** counterfactual thinking, schizophrenia, reasoning, symptomatic remission, illness duration

## Abstract

Counterfactual thinking (CFT) is a type of conditional reasoning involving mental representations of alternatives to past factual events that previous preliminary research has suggested to be impaired in schizophrenia. However, despite the potential impact of these deficits on the functional outcome of these patients, studies examining the role of CFT in this disorder are still few in number. The present study aimed to extent previous results by evaluating CFT in the largest sample to date of schizophrenia patients in symptomatic remission and healthy controls. The relationship with symptomatology, illness duration, and sociodemographic characteristics was also explored.

**Methods:** Seventy-eight schizophrenia patients and 84 healthy controls completed a series of tests that examined the generation of counterfactual thoughts, the influence of the “causal order effect,” and the ability to counterfactually derive inferences by using de Counterfactual Inference Test.

**Results:** Compared with controls, patients generated fewer counterfactual thoughts when faced with a simulated scenario. This deficit was negatively related to scores on all dimensions of the Positive and Negative Syndrome Scale-PANNS, as well as to longer illness duration. The results also showed that schizophrenia patients deviated significantly from the normative pattern when generating inferences from CFT.

**Conclusions:** These findings reveal CFT impairment to be present in schizophrenia even when patients are in symptomatic remission. However, symptomatology and illness duration may have a negative influence on these patients' ability to generate counterfactual thoughts. The results might support the relevance of targeting CFT in future treatment approaches, although further research is needed to better describe the relationship between CFT and both symptomatology and functional outcome.

## Introduction

Counterfactual thinking (CFT) is a specific type of conditional reasoning involving mental representations of alternatives to past situations that were once factual possibilities but which never occurred (Van Hoeck et al., 2015; Byrne, 2016). This process is mainly activated by negative outcomes in the form of “if only” conditional prepositions (Kahneman and Tversky, 1982; Roese, 1997; Byrne and McEleney, 2000). For instance, in the fictional scenario where you have arrived late for work, a counterfactual thought like *If only I had woken up earlier, I would had arrived on time* might be automatically generated.

Being regarded within the *norm theory* (Kahneman and Tversky, 1982) and in the *mental models perspective* (Byrne and McEleney, 2000), CFT appears to play a crucial role supporting adaptive behavior by enabling learning from past experiences (Epstude and Roese, 2008), modulating emotional state (Roese and Olson, 1997), promoting creativity (Markman et al., 2007), and supporting future planning and prediction (Smallman and Roese, 2009). Counterfactual reasoning also serves a behavior-regulating function, influencing behavioral changes and performance improvement (Epstude and Roese, 2008). From the perspective of the cognitive biases tradition, CFT is regarded as enhancing memory distortions that contribute to suboptimal decision-making (Roese and Olson, 1996), as well as being involved in the development of false-belief reasoning (Byrne, 2016). Thus, counterfactual reasoning appears to be a constructive process that requires of the integration of different cognitive functions and psychological processes to construct the internal representations of the target scenario (Van Hoeck et al., 2015). In accordance, although prefrontal cortex (PFC) areas seem to be the primary regions involved (Knight and Grabowecky, 1995; Gomez-Beldarrain et al., 2005), recent studies in healthy control subjects have proposed CFT to depend on an integrative network of systems for affective processing, mental simulation and cognitive control, including both cortical and subcortical structures. This proposal considers that counterfactual reasoning might actually rely on the coordination of multiple information processing systems that together enable adaptive behavior (Barbey et al., 2009; Van Hoeck et al., 2012, 2015).

Regarding its assessment, activation of CFT is generally evaluated by focusing on two aspects: (1) the ability of individuals to spontaneously generate counterfactual alternatives; and (2) the assessment of factors that have been described to influence on the CFT generation, for instance the “causal order effect” (Wells et al., 1987) or the “unusualness” and “proximity” of the situation (Kahneman and Tversky, 1982; Kahneman and Varey, 1990).

Neurocognitive impairment is a core feature of schizophrenia and includes deficits in almost all cognitive domains (Heinrichs and Zakzanis, 1998). This impairment is already observable in the early stages of the disorder (Censits et al., 1997; Keefe et al., 2006b; Crespo-Facorro et al., 2011; Cuesta et al., 2015) and seems to be present even before the initiation of treatment with neuroleptic drugs (Saykin et al., 1994). There is general agreement that neurocognitive performance is a strong correlate of schizophrenia patients' real-world functioning (Green, 1996; Fett et al., 2011) and that psychopathology and cognitive deficits in schizophrenia are probably caused, at least partially, by distinct pathophysiological processes, given that these cognitive deficits are manifested similarly among patients who have attained symptomatic remission and also by those who have not (Green et al., 2004; Buckley et al., 2007; Krishnadas et al., 2007; Brissos et al., 2011). It is still unclear, however, whether this cognitive dysfunction (including non-social, social cognition, and reasoning biases deficits) remains steady, declines, or improves over the course of the illness (Bilder et al., 1992; Censits et al., 1997; Rund, 1998; Harvey et al., 1999; Heaton et al., 2001).

Different symptomatic remission criteria in schizophrenia have been developed over the years to facilitate research and support a positive, longer-term approach to studying outcome in these patients, including neurocognitive functioning (Kane, 2008; Leucht et al., 2008; Levine et al., 2011; Levine and Leucht, 2013). With this objective in mind, Andreasen et al. proposed in 2005 a remission criteria including two components: a symptom-based criterion (low scores on diagnostically relevant symptoms) and a time criterion (duration of 6 months). This criteria, which has been validated and supported by expert authors on this field (van Os et al., 2006; Opler et al., 2007), is defined as a score of ≤3 (mild) on eight selected items of the Positive and Negative Syndrome Scale (PANSS; Kay et al., 1987).

With the aim of adding to knowledge about both brain function in schizophrenia patients and the etiopathogenesis of the disorder, and given that schizophrenia is related to a PFC dysfunction (Goldman-Rakic, 2011), researchers are increasingly exploring counterfactual reasoning in these individuals. As a result of these studies, a general counterfactual reasoning disruption has been described in schizophrenia, including deficits in these patients' ability to generate counterfactual thoughts, in their causality attributing pattern, and in their ability to make counterfactual-derived inferences (Hooker et al., 2000; Contreras et al., 2016). In addition, and alongside other non-social (Sitskoorn et al., 2004; Snitz et al., 2006) and social (Lavoie et al., 2013; Albacete et al., 2016a) cognitive deficits that are shared by first-degree relatives of psychotic patients, difficulties in the generation of counterfactual thoughts have also been observed among non-psychotic first-degree relatives of people with schizophrenia (Albacete et al., 2016b), suggesting that CFT impairment might be a promising candidate cognitive endophenotype for this disorder.

However, although all these findings suggest that CFT disruptions may be a potential target for new psychosocial or pharmacological treatment approaches (Van Hoeck et al., 2015), research on this topic is still scarce. With this objective in mind, the current study reports the assessment of counterfactual reasoning in the largest sample to date of schizophrenia patients in symptomatic remission and healthy control subjects. In accordance with previous research findings of our group, we hypothesize that schizophrenia patients in symptomatic remission will present a poorer performance on all CFT measures explored suggesting the independence of these deficits with symptom severity of the disorder. Using a naturalistic approach, specific objectives include the assessment and comparison between groups of the ability to generate spontaneous counterfactual thoughts, the “causal order effect,” and the ability to make counterfactual-derived inferences. Further associations with symptomatology, illness duration, and sociodemographic characteristics are also explored.

## Materials and methods

### Study design

This case-control study was conducted in the Psychiatry Department of Bellvitge University Hospital, Barcelona, Spain. The Clinical Research Ethics Committee of our hospital approved all study procedures. All subjects gave written informed consent before inclusion.

### Participants

Participants were recruited from the outpatient services of this Psychiatry Department and two associated mental health centers in the same catchment area: the Polyvalent Mental Health Unit—Benito Menni CASM and the Mental Health Unit of L'Hospitalet de Llobregat - Catalan Institute of Health.

Seventy-eight patients who met DSM-IV-TR (American Psychiatric Association, 2000) criteria for schizophrenia were included in the study. None of these patients had undergone electroconvulsive therapy or other brain stimulation therapies (rTMS or TDCS) in the last 6 months, and they all met the criteria for remission as defined by Andreasen et al. (2005): a score of ≤3 (mild) on eight selected items of the PANSS (Kay et al., 1987) (P1, P2, P3, N1, N4, N6, G5, and G9) which is maintained for at least 6 months. Patients whose diagnosis included bipolar, schizoaffective, delusional, or other Axis I disorders were excluded. Eighty-four healthy control subjects were recruited from among hospital employees. In order to be eligible they had to have no history of personal (Axis I and Axis II) or family psychiatric disorder, substance abuse, or suicide attempt.

For both the patient and control groups, additional exclusion criteria were a history of head trauma involving loss of consciousness, an organic disease with mental repercussions, or an estimated intelligence quotient (IQ) below 70. Groups were matched by gender, age, and educational level (measured in years).

### Measures and procedures

Mental and personality disorders were assessed in all potential participants prior to enrolment using the Structured Clinical Interview for DSM-IV Axis I Disorders (SCID-I; First et al., 1997) and Axis II Personality Disorders (SCID-II; First et al., 1994). These individual testing sessions lasted 2 h on average. The clinical rating scales and neurocognitive tests were administered by experienced psychiatrists and psychologists from our team, who also collected the sociodemographic data.

#### Sociodemographic characteristics and clinical assessment

Sociodemographic data was collected for all participants and included age, gender, educational level, and current occupational and civil status. The Vocabulary subtest of the Wechsler Adult Intelligence Scale-III (Wechsler, 1999) was administered to give an estimate IQ that was relatively resistant to postmorbid decline in the patients. The laterality was assessed through the Edinburgh Handedness Inventory (Oldfield, 1971). Psychopathology was assessed using the Spanish adaptation of the Positive and Negative Syndrome Scale (PANSS; Kay et al., 1987; Peralta and Cuesta, 1994). Level of functioning was measured with the Global Assessment of Functioning Scale (GAF; American Psychiatric Association, 2000). The daily dose of antipsychotic medication was calculated in chlorpromazine equivalents (Kane et al., 2003).

#### Assessment of counterfactual thinking

Using set of three different measures, CFT was quantitatively assessed by exploring (1) the spontaneous generation of counterfactual thoughts and (2) some of the factors known to influence it, including the “causal order effect” and the specific characteristics of the situation (such as the “unusualness” or “proximity” of the situation) by using the Counterfactual Inference Test (CIT; Hooker et al., 2000).

On the basis of the research paradigm designed originally by Wells et al. (1987), CFT generation and the “causal order effect” were firstly assessed. This paradigm consists of a scenario involving four consecutive independent events that result in a negative outcome. To avoid first-event bias, the order of the events was randomly changed using a 4 × 4 Latin square design. In the present study, the scenario, which was read aloud to each participant, involved an individual who hears on the radio that a store on the other side of town is offering big price reductions on a limited number of stereo systems. His/her progress in getting there is then impeded by four consecutive minor misfortunes: (a) a speeding ticket, (b) a flat tire, (c) a traffic jam, and (d) a group of elderly people crossing the street. Because of these events, he/she arrives late only to find out that the last stereo system has already been sold just a few minutes earlier. Two experiments were then administered in the following order:

##### Experiment 1: the causal order effect

In this first experiment, participants were asked to choose which one of the four events would they select in order to reverse the scenario. This procedure is based on previous research findings suggesting that CFT tends to be influenced by the order in which the information is presented. Specifically, this effect refers to the tendency among healthy control subjects to choose the first event of a sequence as the most decisive one for the final negative outcome (Wells et al., 1987; Segura et al., 2002). Those participants who were unable to select one event were assigned with the response type “reasoning blocking” to ensure that these answers were not considered as missing data.

##### Experiment 2: generation of counterfactual thoughts

In this second experiment, all participants were asked to say aloud all the possible alternatives they could imagine in order to avoid the final negative outcome of the scenario. These alternatives could be new original ones (e.g., “If only I had called and made a reservation in advance”) or related to one of the misfortunate events (e.g., “If only I hadn't been speeding”). Two independent researchers filtered which answers were real counterfactual thoughts and which ones were illogical or bizarre answers (e.g., “I continued sleeping”).

Finally, the *Counterfactual Inference Test (CIT)*, originally designed by Hooker et al. (2000), was administered to assess the ability to generate counterfactual-derived inferences in front of different hypothetical social situations (for an overview of the test, see Table 1). This self-reporting instrument is based on previous research which has shown not only how specific characteristics of the situation might influence the generation of an inference by enhancing CFT—i.e., situations with outcomes preceded by unusual rather than typical actions (Kahneman and Tversky, 1982) and events that seem “almost” (either spatially or temporally) to have occurred (Kahneman and Varey, 1990), but also how CFT, once activated, can influence the individual's affective and judgmental reactions to the situation (Kahneman and Tversky, 1982; Kahneman and Varey, 1990).

**Table 1:** **The counterfactual inference test (Hooker et al., 2000)**.

**Scenario**	**Response**
1- Reaction of upset (affective) in response to spatial “nearly happened” event *Janet is attacked by a mugger only 10 m from her house. Susan is attacked by a mugger 1 kilometer from her house. Who is more upset by the mugging?*	**a) Janet** b) Susan c) Same/Can't tell
2- Reaction of regret (affective) in response to an “unusual” event *Anna gets sick after eating at a restaurant she often visits. Sarah gets sick after eating at a restaurant she has never visited before. Who regrets their choice of restaurant more?*	a) Anna **b) Sarah** c) Same/Can't tell
3- Reaction of rumination (judgemental) in response to a temporal “nearly happened” event *Jack misses his train by five minutes. Ed misses his train by more than an hour. Who spends more time thinking about the missed train?*	a) Ed **b) Jack** c) Same/Can't tell
4- Reaction of avoidance (judgemental) in response to an “unusual” event *John gets into a car accident while driving on his usual way home. Bob gets into a car accident while trying a new way home. Who thinks more about how his accident could have been avoided?*	**a) Bob** b) John c) Same/Can't tell

*Typical pattern of responses—that is, the target counterfactual responses—are indicated in boldface (Hooker et al., 2000)*.

The CIT presents a set of four scenarios in which two events with similar outcomes are experienced by two different individuals. The circumstances of each pair of events differ such that one of the individuals should think “if only” to a greater extent than the other does. The target questions vary so as to reflect different higher-order inferences: Scenario 1 focuses on a general affective reaction (“upset”) in the context of a spatial “nearly happened” event, Scenario 2 on a general affective reaction (“regret”) in response to an “unusual” event, Scenario 3 on a judgmental or cognitive reaction (“rumination”) brought on by a temporal “nearly happened” event, and Scenario 4 on a judgmental or cognitive reaction (“judgements of avoidance/prevention”) in the face of an “unusual” event. For each scenario, participants are given three possible answers: (1) a target counterfactual response, that is, the option where the subject would most probably think “if only”; (2) a non-target counterfactual response, that is, the option where CFT is also activated but is less likely; and (3) a “same/can't tell” answer, in the event that the participant considers none of the previous options to be suitable. The CIT Total score is calculated from the typical/normative pattern of responses, based on previous research using a sample of undergraduate control subjects (Hooker et al., 2000). Each scenario is given a maximum score of 1 if the subject chooses the normative response, that is, the target counterfactual answer; if the subject chooses any of the other answers the score assigned is zero. Consequently, the total score ranges between 0 and 4, with higher values indicating a response pattern closer to the normative pattern.

### Statistical analysis

Absolute and relative frequencies were calculated for categorical variables, whereas for continuous variables mean (M) and standard deviation (SD) were used for normally distributed variables and the median and interquartile range (IQR) for non-normally distributed variables. Differences between groups where explored by using Fisher's exact test and χ^2^ for categorical data, whereas the *t*-test and the Wilcoxon rank sum test were applied for parametric and non-parametric continuous data, respectively. Normality of distributions was checked using the Kolmogorov-Smirnov test. Multivariate linear regression analysis was used to examine differences between groups in Experiment 2 and in CIT Total score adjusted by age, gender, and estimated IQ. In all analyses, differences were assessed using a statistical test based on two-tailed significance at the 5% level (α = 0.05). Data were managed and analyzed using R 3.1.3.

## Results

### Sociodemographic and clinical characteristics

Sociodemographic and clinical characteristics are presented in Table 2. Compared with control subjects, more schizophrenia patients were single and either unemployed or retired at enrolment, and they also had a lower estimated IQ score.

**Table 2:** **Sociodemographic and clinical characteristics of the sample**.

	**Schizophrenia patients (*n* = 78)**	**Healthy controls (*n* = 84)**	***p*-value**
**SOCIODEMOGRAPHIC CHARACTERISTICS**			
Male gender, *n (%)*	50 (64.1)	45 (53.6)	0.230
Age (years)	40.2 (11.0)	41.7 (12.2)	0.759
Educational level (years)	9.8 (2.9)	10.7 (2.9)	0.050
Employment status, *n (%)*			<0.0001
Employed/Student	18 (23.1)	65 (77.4)	
Retired	39 (50.0)	8 (9.5)	
Unemployed	21 (26.9)	11 (13.1)	
Civil status, *n (%)*			<0.0001
Married	13 (16.7)	40 (47.6)	
Single	31 (36.9)	57 (73.1)	
Divorced	8 (10.3)	10 (11.9)	
Hand Dominance (right), *n (%)*	71 (91)	79 (94)	0.760
Estimated IQ	97.1 (10.7)	107 (10.8)	<0.0001
**CLINICAL MEASURES**			
Age at onset of schizophrenia (years), *median (IQR)*	23.43 (19.0–28.7)		
Duration of the illness (years), *median (IQR)*	15.02 (7.39–24.26)		
Readmissions (episodes), *median (IQR)*	2.00 (1.00–3.00)		
Suicide attempts (none), *n (%)*	60 (76.9)		
GAF	65.2 (7.8)		
Pharmacological treatment^a^, *median (IQR)*	550 (350–888.25)		
PANSS dimensions			
Positive symptoms	13.26 (3.06)		
Negative symptoms	20.75 (4.60)		
General psychopathology	34.92 (7.28)		
Total score	68.9 (13.1)		

*Values presented as means (standard deviation) unless otherwise specified. IQR, interquartile range; GAF, global assessment of functioning; PANSS, positive and negative syndrome scale*.

a *Milligrams per day of chlorpromazine equivalents*.

### Experiment 1: the causal order effect

No differences were observed between groups when choosing one event as being the most relevant for reversing the scenario (*p* = 0.197); in other words, the general pattern of response was not different between groups. Specifically, results showed that both patients and controls tended to choose the first event as the main determinant. The proportion of participants unable to choose any of the events (i.e., the “reasoning blocking” answer) was not significantly different between the two groups (*p* = 0.078).

### Experiment 2: generation of counterfactual thoughts

**Figure 1:**
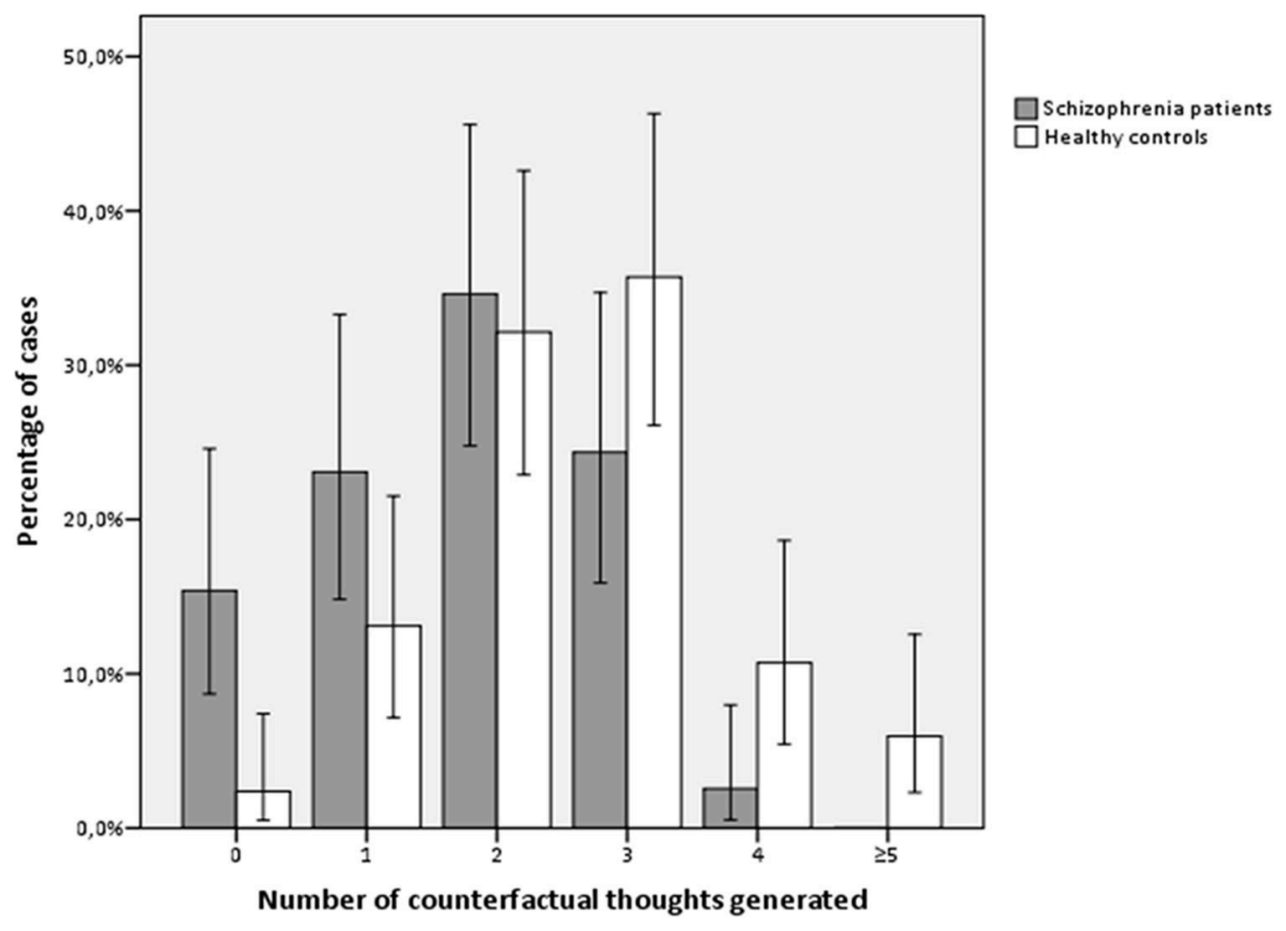
**Number of counterfactual thoughts generated by groups of study (Experiment 2)**.

Schizophrenia patients in symptomatic remission not only generated significantly fewer spontaneous alternatives (including both real and non-real counterfactual thoughts, *p* = 0.000) but also fewer counterfactual thoughts in comparison with healthy controls (*p* < 0.0001). These significant differences were independent of gender, age, and estimated IQ (*p* < 0.0001; Figure 1).

### CIT: generation of counterfactually derived inferences

Two significant differences were found between groups on the CIT (Table 3): whereas in Scenario 1 (upset in a spatial “nearly happened” event) patients chose the target-counterfactual answer more frequently (*p* = 0.011), in Scenario 3 (rumination in a temporal “nearly happened” event) the group of schizophrenia patients chose the target-counterfactual response significantly less often than did controls (*p* = 0.034). However, there was no statistically significant difference between the groups in CIT Total score (*p* = 0.594).

**Table 3:** **CIT results: descriptive and comparative analysis of the counterfactually derived inferences assessment**.

	**Schizophrenia patients (*n* = 78)**	**Healthy controls (*n* = 84)**	***p*-value**
Total score, *n (%)*			0.594
0	8 (10.4)	7 (8.3)	
1	17 (22.1)	18 (21.4)	
2	27 (35.1)	29 (34.5)	
3	19 (24.7)	22 (26.2)	
4	6 (7.8)	8 (9.5)	
Total score, *median (IQR)*	2.00 (1.00–3.00)	2.00 (1.00–3.00)	
Scenario 1: *Upset in spatial “nearly happened” event, n (%)*			0.011
Target counterfactual response	29 (37.7)	22 (26.2)	
Non-target counterfactual response	15 (19.5)	7 (8.3)	
Same/can't tell	33 (42.9)	55 (65.5)	
Scenario 2: *Regret in unusual event, n (%)*			0.150
Target counterfactual response	34 (44.2)	50 (59.5)	
Non-target counterfactual response	22 (28.6)	17 (20.2)	
Same/can't tell	21 (27.3)	17 (20.2)	
Scenario 3: *Rumination in temporal “nearly happened” event, n (%)*			0.034
Target counterfactual response	45 (58.4)	59 (70.2)	
Non-target counterfactual response	24 (31.2)	12 (14.3)	
Same/can't tell	8 (10.4)	13 (15.5)	
Scenario 4: *Judgements of avoidance in unusual event, n (%)*			0.840
Target counterfactual response	43 (55.8)	43 (51. 2)	
Non-target counterfactual response	16 (20.8)	19 (22.6)	
Same/can't tell	18 (23.4)	22 (26.2)	

*IQR, interquartile range*.

### CFT and sociodemographic and clinical measures in schizophrenia patients

The results in Table 4 suggest that among schizophrenia patients a less frequent generation of counterfactual thoughts (Experiment 2) was negatively associated with all PANSS dimensions (Positive symptoms, *p* = 0.007; Negative symptoms, *p* = 0.015; General symptoms, *p* = 0.050; Total score, *p* = 0.011) and with more than 10 years of illness duration (*p* = 0.028), with this analysis being adjusted by gender, age, and estimated IQ. However, no significant associations were found between performance in Experiment 1 or on the CIT and any of the clinical measures examined, including the daily dose of antipsychotics. None of the sociodemographic characteristics were related to performance on any of the CFT tasks administered.

**Table 4:** **Multivariate linear regression analysis to evaluate clinic factors associated with the generation of counterfactual thoughts among schizophrenia patients**.

**Schizophrenia patients**	Coeff^*^	***t*-value**	***p*-value**
Duration of illness (years)	−0.041	−1.857	0.067
<10 years vs. ≥10 years	−0.746	−2.243	0.028
Readmissions			
None vs. 1 episode	−0.008	−0.021	0.983
None vs. 2 episodes	0.200	0.469	0.641
None vs. 3 episodes	0.364	0.908	0.367
Suicide attempts (number of episodes)			
None vs. at least one	−0.087	−0.294	0.770
GAF	0.002	−0.114	0.909
Pharmacological treatment	−0.000	−0.591	0.556
PANSS dimensions			
Positive symptoms	−0.104	−2.783	0.007
Negative symptoms	−0.064	−2.497	0.015
General psychopathology	−0.032	−1.995	0.050
Total score	−0.023	−2.606	0.011

*GAF, global assessment of functioning; PANSS, positive and negative syndrome scale; Coeff, coefficients*.

* *Adjusted by age, gender, and estimated IQ*.

## Discussion

The present study extends previous results of our group by reporting the assessment of counterfactual reasoning in the largest sample to date of schizophrenia patients in symptomatic remission and healthy controls. In addition, potential associations with sociodemographic characteristics, symptomatology, and illness duration have been further examined. As a result, the main finding of the study is that, compared with controls, schizophrenia patients with symptomatic remission significantly generate fewer counterfactual thoughts when faced with a simulated scenario. Regarding the effect of external factors influencing CFT, results also suggest a significant deviation from the normative pattern among patients when counterfactually deriving inferences. However, no significant differences are found regarding the causal attribution pattern. Finally, among patients, deficits in the CFT generation appear to be negatively related to scores on all dimensions of the PANNS, as well as to longer duration of illness.

Compared to what is normally expected in the general population, patients with schizophrenia in a state of symptomatic remission seem to generate significantly fewer spontaneous alternative representations using CFT in the face of a fictional situation with a negative outcome. These findings reinforce the hypothesis that schizophrenia is a mental condition in which patients have difficulties in using conditional reasoning to re-imagine a negative outcome in a positive way, a process that enables the activation of alternative representations for dealing with reality. Alongside recent findings among unaffected first-degree relatives of schizophrenia patients (Albacete et al., 2016b), the present results also add to our knowledge of cognitive deficits as core features of schizophrenia since these deficits do not seem to be simply the result of symptoms or the pharmacological treatments administered for the illness—i.e., do not seem to share the same underlying pathological process causing the clinical symptoms of the disorder (Gold, 2004; Green et al., 2004).

Interestingly, our data analyses also reveal significant negative associations between CFT generation and scores on the PANSS, including total score and all three symptom dimensions. These results indicate that, although on average PANSS scores were mild, those patients with greater symptom severity tend to generate fewer counterfactual alternatives. This suggests that although these deficits might not be the result of symptoms, they may be influenced by them. This would be in line with previous literature that has related classic symptomatology of the disorder to other neurocognitive deficits (Addington et al., 1991; Shurman et al., 2005; Keefe et al., 2006a).

The results of this experiment also show that schizophrenia patients with longer illness duration are those who tend to generate fewer counterfactual thoughts, suggesting that this deficit does not settle into a stable pattern but, rather, tends to deteriorate over time in schizophrenia. This would be contrary to previous research indicating that cognitive impairment, including non-social, social cognition, and reasoning biases, remains stable over time regardless of changes in clinical state (Heaton et al., 1994, 2001; Rund, 1998; Peters and Garety, 2006; Horan et al., 2012). Our findings therefore appear to warrant further investigation, for instance, by conducting follow-up studies.

Regarding the ability to counterfactually derive inferences, results of the CIT suggest that, compared with controls, the schizophrenia patients react with greater upset in the face of a spatial “nearly happened” event (Scenario 1). Whereas from a clinical point of view this stronger general affective reaction might be related to positive symptoms of the illness such as hostility, from a functional point of view this biased inference generation would lead to difficulties in these patients' ability to deal with interpersonal relationships.

Our analyses also revealed that in comparison with controls, schizophrenia patients react significantly less often with rumination in the face of a temporal “nearly happened” event (Scenario 3). Consistent with previous results reported by our group (Contreras et al., 2016), this tendency to disregard the negative outcome of a social event might be related to negative symptomatology such as blunted affect or emotional withdrawal. This diminished reaction might also be associated with poor psychosocial and vocational functioning among these patients.

Furthermore, the analysis of CIT Total score suggests that schizophrenia patients and healthy controls perform similarly when deriving inferences from CFT. However, it should be noted that the total score of 2/4 found in healthy controls is not consistent with the normative pattern (i.e., a total score of 4/4) originally proposed by Hooker et al. (2000). In fact, previous studies by our group also failed to observe a score of 4/4 among controls, with these subjects tending to score 2 or 3 instead (Albacete et al., 2016b; Contreras et al., 2016). Thus, given that the pattern of response differs significantly between studies not only on total score but also for each scenario, further research is required to elucidate the role of CFT in the generation of cognitive inferences. Importantly, this would entail revising the CIT design and improving test reliability, for instance, by extending the number of scenarios.

Finally, results of the causal order effect' experiment show that influences CFT similarly in both groups, in other words, the response pattern when deciding which of the events is more decisive does not differ between patients and controls. Moreover, and as observed in the general population (Wells et al., 1987), both groups tend to choose the first event as being the most determinant. Contradicting previous findings in a smaller sample of schizophrenia patients who did not meet criteria for symptomatic remission (Contreras et al., 2016), the present results might be cause for optimism since they indicate a normative pattern of causality attribution in schizophrenia.

The present study has a number of shortcomings that should be acknowledged. First, although the number of participants is greater than in previous studies exploring CFT in schizophrenia, an even larger sample might have achieved better statistical power. Second, the case-control design prevents us from drawing conclusions as to whether the CFT impairment observed originates after or before the onset of the illness, although it should be noted that in order to avoid a potential effect of greater cognitive deterioration among older schizophrenia patients, healthy subjects were matched by age, gender, and years of education.

In conclusion, the present study seems to confirm the presence in schizophrenia of impairment in the ability to generate spontaneous counterfactual thoughts. This deficit is present despite the fact that patients are in symptomatic remission, suggesting the independence of this deficit alongside other reported neurocognitive deficits in schizophrenia (i.e., the findings support the consideration of cognitive impairment as being a core feature of the disorder). However, the present results also suggest that clinical factors such as classic symptomatology and illness duration might have a negative impact on the ability to generate counterfactual thoughts. Longitudinal studies might therefore be warranted in order to extend and confirm these findings. It should also be noted that schizophrenia patients deviated from the normative pattern when deriving inferences from CFT in situations involving reactions of regret and rumination in the face of a “nearly happened” event. Finally, the results also suggest that the causal attribution pattern is preserved among these patients.

Given the potential ecological impact that impaired counterfactual reasoning may have on the day-to-day functioning of these patients (Roese and Olson, 1997), a disruption in the generation of counterfactual thoughts and inferences derived from CFT might be considered a new putative target for future psychosocial or even pharmacological treatment approaches. Further research is required to better describe how these deficits are related to symptomatology and functional outcome in this disorder, for instance by developing new tools of counterfactual inference assessment. Finally, it would also be interesting to explore whether the proposed integrative network of systems supporting CFT is disrupted in schizophrenia by using neuroimaging techniques.

## Ethics statement

This study was carried out in accordance with the recommendations of the Clinical Research Ethics Committee of the Bellvitge University Hospital with written informed consent from all subjects. All subjects gave written informed consent in accordance with the Declaration of Helsinki. The protocol was approved by the Clinical Research Ethics Committee of the Bellvitge University Hospital.

## Author contributions

AA and FC contributed to the management of the literature searches, design of the study, carried out the cognitive explorations and undertook the statistical analysis. CB, EG, and ÁA contributed in the sample recruitment and psychopathological evaluations. JM supervised the data collection, contributed to the management of the literature searches and assisted with study design. All authors participated in the writing process, read and approved the final manuscript, and are in agreement to be accountable for all aspects of the work in ensuring that questions related to the accuracy or integrity of any part of the work are appropriately investigated and resolved.

## Funding

This work was supported by the Spanish Ministry of Science and Innovation, the Carlos III Health Institute (PI11/02221) and the University of Barcelona (AA, APIF-UB grants).

### Conflict of interest statement

The authors declare that the research was conducted in the absence of any commercial or financial relationships that could be construed as a potential conflict of interest.

## References

[B1] Addington J. Addington D. Maticka-Tyndale E. (1991). Cognitive functioning and positive and negative symptoms in schizophrenia. Schizophr. Res. 5, 123–134. 10.1016/0920-9964(91)90039-T 1931805

[B2] Albacete A. Bosque C. Custal N. Crespo J. M. Gilabert E. Albiach A.. (2016a). Emotional intelligence in non-psychotic first-degree relatives of people with schizophrenia. Schizophr. Res. 175, 103–108. 10.1016/j.schres.2016.04.039 27177808

[B3] Albacete A. Contreras F. Bosque C. Gilabert E. Albiach Á. Menchón J. M.. (2016b). Counterfactual reasoning in non-psychotic first-degree relatives of people with schizophrenia. Front. Psychol. 7:665. 10.3389/fpsyg.2016.00665 27242583 PMC4860705

[B4] American Psychiatric Association (2000). Diagnostic and Statistical Manual of Mental Disorders, 4th Edn. Washington, DC: APA.

[B5] Andreasen N. C. Carpenter W. T. Kane J. M. Lasser R. A. Marder S. R. Weinberger D. R. (2005). Remission in schizophrenia: proposed criteria and rationale for consensus. Am. J. Psychiatry 162, 441–449. 10.1176/appi.ajp.162.3.441 15741458

[B6] Barbey A. K. Krueger F. Grafman J. (2009). Structured event complexes in the medial prefrontal cortex support counterfactual representations for future planning. Philos. Trans. R. Soc. Lond. B. Biol. Sci. 364, 1291–1300. 10.1098/rstb.2008.0315 19528010 PMC2666713

[B7] Bilder R. M. Lipschutz-Broch L. Reiter G. Geisler S. H. Mayerhoff D. I. Lieberman J. A. (1992). Intellectual deficits in first-episode schizophrenia: evidence for progressive deterioration. Schizophr. Bull. 18, 437–448. 10.1093/schbul/18.3.437 1411331

[B8] Brissos S. Dias V. V. Balanzá-Martinez V. Carita A. I. Figueira M. L. (2011). Symptomatic remission in schizophrenia patients: relationship with social functioning, quality of life, and neurocognitive performance. Schizophr. Res. 129, 133–136. 10.1016/j.schres.2011.04.001 21514793

[B9] Buckley P. F. Harvey P. D. Bowie C. R. Loebel A. (2007). The relationship between symptomatic remission and neuropsychological improvement in schizophrenia patients switched to treatment with ziprasidone. Schizophr. Res. 94, 99–106. 10.1016/j.schres.2006.12.032 17499480

[B10] Byrne R. M. (2016). Counterfactual Thought. Annu. Rev. Psychol. 67, 135–157. 10.1146/annurev-psych-122414-033249 26393873

[B11] Byrne R. M. McEleney A. (2000). Counterfactual thinking about actions and failures to act. J. Exp. Psychol. 26, 1318–1331. 10.1037/0278-7393.26.5.1318 11009260

[B12] Censits D. M. Ragland J. D. Gur R. C. Gur R. E. (1997). Neuropsychological evidence supporting a neurodevelopmental model of schizophrenia: a longitudinal study. Schizophr. Res. 24, 289–298. 10.1016/S0920-9964(96)00091-6 9134589 PMC4334367

[B13] Contreras F. Albacete A. Castellví P. Caño A. Benejam B. Menchón J. M. (2016). Counterfactual reasoning deficits in schizophrenia patients. PLoS ONE 11:e148440. 10.1371/journal.pone.0148440 26828931 PMC4734710

[B14] Crespo-Facorro B. Roiz-Santiá-ez R. Pérez-Iglesias R. Rodriguez-Sanchez J. M. Mata I. Tordesillas-Gutierrez D.. (2011). Global and regional cortical thinning in first-episode psychosis patients: relationships with clinical and cognitive features. Psychol. Med. 41, 1449–1460. 10.1017/S003329171000200X 20942995 PMC3954972

[B15] Cuesta M. J. Sánchez-Torres A. M. Cabrera B. Bioque M. Merchán-Naranjo J. Corripio I.. (2015). Premorbid adjustment and clinical correlates of cognitive impairment in first-episode psychosis. The PEPsCog Study. Schizophr. Res. 164, 65–73. 10.1016/j.schres.2015.02.022 25819935

[B16] Epstude K. Roese N. J. (2008). The functional theory of counterfactual thinking. Personal. Soc. Psychol. Rev. 12, 168–192. 10.1177/1088868308316091 18453477 PMC2408534

[B17] Fett A. K. J. Viechtbauer W. Dominguez M.-G. Penn D. L. Van Os J. Krabbendam L. (2011). The relationship between neurocognition and social cognition with functional outcomes in schizophrenia: a meta-analysis. Neurosci. Biobehav. Rev. 35, 573–588. 10.1016/j.neubiorev.2010.07.001 20620163

[B18] First M. B. Spitzer R. L. Gibbon M. Williams J. B. W. (1997). Structured Clinical Interview for DSM-IV Axis I Disorders—Clinician Version (SCID-CV). Washington, DC: American Psychiatric Press.

[B19] First M. B. Spitzer R. L. Gibbon M. Williams J. W. B. Benjamin L. (1994). Structured Clinical Interview for DSM-IV Axis II Personality Disorders (SCID-II). New York, NY: New York State Psychiatric Institute.

[B20] Gold J. M. (2004). Cognitive deficits as treatment targets in schizophrenia. Schizophr. Res. 72, 21–28. 10.1016/j.schres.2004.09.008 15531404

[B21] Goldman-Rakic P. S. (2011). Circuitry of primate prefrontal cortex and regulation of behavior by representational memory, in Comprehensive Physiology, ed American Physiological Society (New York, NY: American Physiological Society), 373–417.

[B22] Gomez-Beldarrain M. Garcia-Monco J. C. Astigarraga E. Gonzalez A. Grafman J. (2005). Only spontaneous counterfactual thinking is impaired in patients with prefrontal cortex lesions. Cogn. Brain Res. 24, 723–726. 10.1016/j.cogbrainres.2005.03.013 16099374

[B23] Green M. (1996). What are the functional consequences of neurocognitive deficits in schizophrenia? Am. J. Psychiatry 153, 321–330. 10.1176/ajp.153.3.321 8610818

[B24] Green M. F. Nuechterlein K. H. Gold J. M. Barch D. M. Cohen J. Essock S.. (2004). Approaching a consensus cognitive battery for clinical trials in schizophrenia: the NIMH-MATRICS conference to select cognitive domains and test criteria. Biol. Psychiatry 56, 301–307. 10.1016/j.biopsych.2004.06.023 15336511

[B25] Harvey P. D. Silverman J. M. Mohs R. C. Parrella M. White L. Powchik P.. (1999). Cognitive decline in late-life schizophrenia: a longitudinal study of geriatric chronically hospitalized patients. Biol. Psychiatry 45, 32–40. 10.1016/S0006-3223(98)00273-X 9894573

[B26] Heaton R. K. Gladsjo J. A. Palmer B. W. Kuck J. Marcotte T. D. Jeste D. V. (2001). Stability and course of neuropsychological deficits in schizophrenia. Arch. Gen. Psychiatry 58, 24–32. 10.1001/archpsyc.58.1.24 11146755

[B27] Heaton R. Paulsen J. S. McAdams L. A. Kuck J. Zisook S. Braff D.. (1994). Neuropsychological deficits in schizophrenics. Arch. Gen. Psychiatry 51, 469–476. 10.1001/archpsyc.1994.03950060033003 8192549

[B28] Heinrichs R. W. Zakzanis K. K. (1998). Neurocognitive deficit in schizophrenia: a quantitative review of the evidence. Neuropsychology 12, 426–445. 10.1037/0894-4105.12.3.426 9673998

[B29] Hooker C. Roese N. J. Park S. (2000). Impoverished counterfactual thinking is associated with schizophrenia. Psychiatry 63, 326–335. 10.1080/00332747.2000.11024925 11218555

[B30] Horan W. P. Green M. F. DeGroot M. Fiske A. Hellemann G. Kee K.. (2012). Social cognition in schizophrenia, Part 2: 12-month stability and prediction of functional outcome in first-episode patients. Schizophr. Bull. 38, 865–872. 10.1093/schbul/sbr001 21382881 PMC3406537

[B31] Kahneman D. Tversky A. (1982). The simulation heuristic, in Judgment Under Uncertainty: Heuristics and Biases, eds Kahneman D. Slovic P. Tversky A. (Cambridge: Cambridge University Press), 201–208.

[B32] Kahneman D. Varey C. A. (1990). Propensities and counterfactuals: the loser that almost won. J. Pers. Soc. Psychol. 59, 1101–1110. 10.1037/0022-3514.59.6.1101

[B33] Kane J. M. (2008). An evidence-based strategy for remission in schizophrenia. J. Clin. Psychiatry 69(Suppl. 3), 25–30. 18533759

[B34] Kane J. M. Leucht S. Carpenter D. Docherty J. (2003). Optimizing pharmacological treatment of psychotic disorders. J. Clin. Psychiatry 64, 21–51. 14640142

[B35] Kay S. R. Fiszbein A. Opler L. A. (1987). The positive and negative syndrome scale (PANSS) for schizophrenia. Schizophr. Bull. 13, 261–276. 10.1093/schbul/13.2.261 3616518

[B36] Keefe R. S. E. Bilder R. M. Harvey P. D. Davis S. M. Palmer B. W. Gold J. M.. (2006a). Baseline neurocognitive deficits in the CATIE schizophrenia trial. Neuropsychopharmacology 31, 2033–2046. 10.1038/sj.npp.1301072 16641947

[B37] Keefe R. S. Perkins D. O. Gu H. Zipursky R. B. Christensen B. K. Lieberman J. A. (2006b). A longitudinal study of neurocognitive function in individuals at-risk for psychosis. Schizophr. Res. 88, 26–35. 10.1016/j.schres.2006.06.041 16930949

[B38] Knight R. T. Grabowecky M. (1995). Escape from linear time: prefrontal cortex and conscious experience, in The Cognitive Neurosciences, ed Gazzaniga M. S. (Cambridge, MA: MIT Press), 1357–1371.

[B39] Krishnadas R. Moore B. P. Nayak A. Patel R. R. (2007). Relationship of cognitive function in patients with schizophrenia in remission to disability: a cross-sectional study in an Indian sample. Ann. Gen. Psychiatry 6:19. 10.1186/1744-859x-6-19 17663763 PMC1976613

[B40] Lavoie M. A. Plana I. Bédard Lacroix J. Godmaire-Duhaime F. Jackson P. L. Achim A. M. (2013). Social cognition in first-degree relatives of people with schizophrenia: a meta-analysis. Psychiatry Res. 209, 129–135. 10.1016/j.psychres.2012.11.037 23375626

[B41] Leucht S. Shamsi S. A. R. Busch R. Kissling W. Kane J. M. (2008). Predicting antipsychotic drug response - Replication and extension to six weeks in an international olanzapine study. Schizophr. Res. 101, 312–319. 10.1016/j.schres.2008.01.018 18308513

[B42] Levine S. Z. Leucht S. (2013). Attaining and sustaining remission of predominant negative symptoms. Schizophr. Res. 143, 60–64. 10.1016/j.schres.2012.11.010 23218563

[B43] Levine S. Z. Rabinowitz J. Ascher-svanum H. Faries D. E. Lawson A. H. (2011). Extent of attaining and maintaining symptom remission by antipsychotic medication in the treatment of chronic schizophrenia: evidence from the CATIE study. Schizophr. Res. 133, 42–46. 10.1016/j.schres.2011.09.018 22000938

[B44] Markman K. D. Lindberg M. J. Kray L. J. Galinsky A. D. (2007). Implications of counterfactual structure for creative generation and analytical problem polving. Personal. Soc. Psychol. Bull. 33, 312–324. 10.1177/0146167206296106 17312314

[B45] Oldfield R. C. (1971). The assessment and analysis of handedness: the Edinburgh inventory. Neuropsychologia 9, 97–113. 10.1016/0028-3932(71)90067-4 5146491

[B46] Opler M. G. Yang L. H. Caleo S. Alberti P. (2007). Statistical validation of the criteria for symptom remission in schizophrenia: preliminary findings. BMC Psychiatry 7:35. 10.1186/1471-244X-7-35 17650312 PMC1949820

[B47] Peralta V. Cuesta M. J. (1994). Validación de la escala de síntomas positivos y negativos (PANSS) en una muestra de esquizofrénicos espa-oles. Actas Luso Espa-olas Neurol. Psiquiátrica 4, 44–50. 7810373

[B48] Peters E. Garety P. (2006). Cognitive functioning in delusions: a longitudinal analysis. Behav. Res. Ther. 44, 481–514. 10.1016/j.brat.2005.03.008 15913544

[B49] Roese N. J. (1997). Counterfactual thinking. Psychol. Bull. 121, 133–148. 9000895 10.1037/0033-2909.121.1.133

[B50] Roese N. J. Olson J. M. (1996). Counterfactuals, causal attributions, and the hindsight bias: a conceptual integration. J. Exp. Soc. Psychol. 32, 197–227. 10.1006/jesp.1996.0010

[B51] Roese N. J. Olson J. M. (1997). Counterfactual thinking: the intersection of affect and function. Adv. Exp. Soc. Psychol. 29, 1–59. 10.1016/S0065-2601(08)60015-5

[B52] Rund B. (1998). A review of longitudinal studies of cognitive functions in schizophrenia patients. Schizophr. Bull. 24, 425–435. 10.1093/oxfordjournals.schbul.a033337 9718634

[B53] Saykin A. J. Shtasel D. L. Gur R. E. Kester D. B. Mozley L. H. Stafiniak P. (1994). Neuropsychological deficits in neuroleptic naive patients with first episode schizophrenia. Arch. Gen. Psychiatry 51, 124–131. 10.1001/archpsyc.1994.03950020048005 7905258

[B54] Segura S. Fernandez-Berrocal P. Byrne R. M. (2002). Temporal and causal order effects in thinking about what might have been. Q. J. Exp. Psychol. A. 55, 1295–1305. 10.1080/02724980244000125 12420996

[B55] Shurman B. Horan W. P. Nuechterlein K. H. (2005). Schizophrenia patients demonstrate a distinctive pattern of decision-making impairment on the Iowa Gambling Task. Schizophr. Res. 72, 215–224. 10.1016/j.schres.2004.03.020 15560966

[B56] Sitskoorn M. M. Aleman A. Ebisch S. J. Appels M. C. Kahn R. S. (2004). Cognitive deficits in relatives of patients with schizophrenia: a meta-analysis. Schizophr. Res. 71, 285–295. 10.1016/j.schres.2004.03.007 15474899

[B57] Smallman R. Roese N. J. (2009). Counterfactual thinking facilitates behavioral intentions. J. Exp. Soc. Psychol. 45, 845–852. 10.1016/j.jesp.2009.03.002 20161221 PMC2717727

[B58] Snitz B. E. Macdonald A. W. Carter C. S. (2006). Cognitive deficits in unaffected first-degree relatives of schizophrenia patients: a meta-analytic review of putative endophenotypes. Schizophr. Bull. 32, 179–194. 10.1093/schbul/sbi048 16166612 PMC2632195

[B59] Van Hoeck N. Ma N. Ampe L. Baetens K. Vandekerckhove M. Van Overwalle F. (2012). Counterfactual thinking: an fMRI study on changing the past for a better future. Soc. Cogn. Affect. Neurosci. 8, 556–564. 10.1093/scan/nss031 22403155 PMC3682438

[B60] Van Hoeck N. Watson P. D. Barbey A. K. (2015). Cognitive neuroscience of human counterfactual reasoning. Front. Hum. Neurosci. 9:420. 10.3389/fnhum.2015.00420 26257633 PMC4511878

[B61] van Os J. Drukker M. Campo J. À. Meijer J. Bak M. Delespaul P. (2006). Validation of remission criteria for schizophrenia. Am. J. Psychiatry 163, 2000–2002. 10.1176/ajp.2006.163.11.2000 17074953

[B62] Wechsler D. (1999). Wechsler Adults Intelligence Scale III. Madrid: TEA Ediciones.

[B63] Wells G. L. Taylor B. R. Turtle J. W. (1987). The undoing of scenarios. J. Pers. Soc. Psychol. 53, 421–430. 10.1037/0022-3514.53.3.421

